# Enhancing Viability in Static and Perfused 3D Tissue Constructs Using Sacrificial Gelatin Microparticles

**DOI:** 10.1021/acsbiomaterials.4c02169

**Published:** 2025-04-07

**Authors:** Andrew R. Hudson, Daniel J. Shiwarski, Alec J. Kramer, Adam W. Feinberg

**Affiliations:** † Department of Biomedical Engineering, Carnegie Mellon University, Pittsburgh, Pennsylvania 15213, United States; ‡ Department of Materials Science & Engineering, Carnegie Mellon University, Pittsburgh, Pennsylvania 15213, United States

**Keywords:** perfusion, scaffold, tissue engineering, bioprinting, porogen, viability

## Abstract

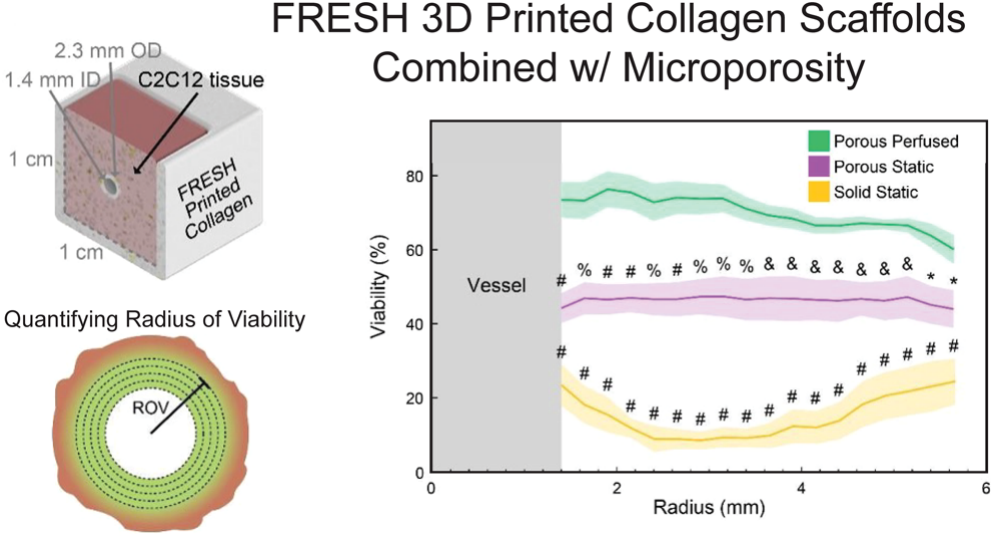

Current limitations in engineered tissues arise from the inability to provide sufficient nutrients to cells deep within constructs, restricting their viability. This study focuses on enhancing diffusion by creating a microporous microenvironment using gelatin microparticles within collagen scaffolds. By leveraging the FRESH (Freeform Reversible Embedding of Suspended Hydrogels) 3D bioprinting technique, gelatin microparticles are utilized both as a support material and as a thermoresponsive porogen to establish interconnected pores. The results indicate that scaffolds with 75% porosity significantly increase diffusion rates and cell viability, extending beyond the conventional ∼200 μm limit. Additionally, integrating vascular-like channels with porous scaffolds and applying perfusion improved nutrient transport, leading to enhanced cell survival in larger constructs. This combination of microporosity and perfusion represents a promising approach to create thicker tissues without necrotic regions, potentially paving the way for scalable tissue engineering applications. The findings suggest that optimizing pore sizes and scaffold perfusion can bridge the gap between rapid tissue formation and slower vascularization processes, enabling the future development of functional tissue constructs at clinically relevant scales.

## Introduction

Vascularization remains a foundational challenge in the tissue engineering field, with the goal to be able to provide mass transport of nutrients and removal of waste to all cells within an engineered construct. Currently, the limit in the size of tissue constructs that can be engineered is not due to the limits of cell production, but rather the inability to provide all cells with sufficient nutrients upon tissue formation, resulting in necrosis of the tissue core. It is commonly stated that cells need to be within 200 μm of a blood vessel in order to survive and function, but this can vary widely depending on cell and tissue type, with capillaries spaced ∼20 μm in the myocardium where cardiomyocytes contract continuously and capillaries being entirely absent in articular cartilage and the cornea where low cell density and diffusion through the collagen-rich tissue is adequate over 500–1000 μm. In vivo the problem of necrotic cores in developing tissues is minimal, as a tissue will naturally form in concert with its microvasculature. In vitro, however, organoids and larger tissue constructs can be formed in minutes to days, outpacing the rate at which microvasculature can naturally form by orders of magnitude. The result of this disparity is the formation of hypoxic and ultimately necrotic regions where only cells within ∼200 μm of the tissue surface or a perfusable channel can rely on passive nutrient diffusion to maintain viability. Since microvasculature cannot currently be created at the same rate one can engineer a bulk tissue, microvasculature growth remains a rate-limiting step in tissue engineering.

While vascularization is essential for the viability of most engineered tissues, alternative strategies for mass transport can be employed to bridge the gap before sufficient vascular growth occurs. One such strategy focuses on microporosity, which plays a significant role in modulating cellular behaviors by providing a more favorable microenvironment. Key parameters that influence its effectiveness include the size of the pores and the percentage of void space within the scaffold.^1−8^ These factors directly impact nutrient diffusion, enabling cells embedded within the construct to receive necessary nutrients and oxygen more effectively.^9^ Additionally, microporosity facilitates cellular infiltration, promoting deeper penetration of cells into the scaffold, which is crucial for the development of functional tissues.^10^ When implanted in vivo, porous scaffolds have been shown to enhance angiogenesis, thereby improving integration with host tissues.^6,8,11^ Research has demonstrated that a variety of fabrication techniques can be utilized to create porous constructs, each offering unique advantages. Methods such as salt leaching,^12^ freeze-drying,^13^ gas foaming, and electrospinning^14,15^ have been extensively explored to achieve controlled porosity. These techniques allow for the fine-tuning of pore architecture to optimize tissue integration and nutrient transport. The primary objective behind increasing the porosity of tissue scaffolds is to reach a critical threshold of interconnectivity between pores. When this critical porosity is achieved, the interconnected pore network significantly enhances the diffusion rates of essential molecules, such as oxygen and nutrients, throughout the matrix. This increase in diffusion is crucial for preventing hypoxia and necrosis within the core of engineered tissues, thus extending the viable thickness of the constructs. Ultimately, optimizing porosity not only supports initial cell survival but also provides a more robust foundation for subsequent vascularization and long-term tissue functionality.

Here we investigate strategies to address the problem of necrotic core formation due to insufficient nutrient mass transport in cell-laden hydrogel scaffolds. By engineering a porous cellular microenvironment, the goal is to increase the typical diffusion distance beyond ∼200 μm, thereby increasing cell viability further into the construct volume. While prevascularized beds and vascularized scaffolds address the challenge of supporting larger tissue constructs, our strategy serves as a transitional solution, bridging the gap until vascularization is formed and matures, and is advantageous for applications requiring immediate nutrient diffusion. By integrating enhanced porosity, our method is intended to mitigate the constraints associated with passive diffusion in early tissue culture stages. This has the potential to integrate synergistically with existing nutrient delivery strategies, offering a complementary enhancement to current approaches. To achieve this control of microporosity, we were inspired by our prior research in 3D bioprinting using the freeform reversible embedding of suspended hydrogels (FRESH) technique.^16^ In FRESH, bioinks such as alginate, fibrin, and collagen type I are extruded within a sacrificial support bath composed of gelatin microparticles compacted into a slurry.^11,17^ FRESH printed objects are innately porous as the gelatin microparticles comprising the support bath are incorporated into the embedded ink and are subsequently melted out during print release.^11,17^ This inherent porosity significantly increases cellular and vessel infiltration both in vitro and in vivo.^11,18^ Here, an alternative use of the FRESH gelatin microparticles is investigated as a thermoresponsive scaffold porogen. First, the gelatin microparticles are used to increase the rate at which nutrients can passively diffuse through hydrogel constructs, achieving interconnected micropore networks that can serve as a rudimentary substitute for a microvessel network, allowing nutrients to diffuse more rapidly under static culture conditions. Second, this approach is combined with FRESH 3D bioprinting to create a cell-laden tissue construct around a perfused collagen-walled vessel. This provides assessment of how media delivered directly to the center of a tissue construct can perfuse out into the bulk hydrogel scaffold under an applied pressure gradient that can increase convection and diffusion. Overall, this study demonstrates how microporosity and engineered vascular-like channels can be combined to minimize the formation of necrotic cores, providing a path forward for engineering larger tissue constructs.

## Materials and Methods

### Fabrication and Analysis of Porous Collagen Scaffolds

Collagen scaffolds of increasing porosity were generated by mixing gelatin microparticles into a collagen solution during the gelation process and then melting the gelatin to leave behind defined pores. To make scaffolds of 0% porosity, 70 mg/mL acidified collagen type I bioink (LifeInk 220, Advanced Biomatrix, 5343) was mixed with deionized water and 10× PBS using a positive displacement pipet to create a 10 mg/mL acidified collagen, 1× PBS solution. The 1 M NaOH solution was then added and rapidly mixed to neutralize the solution with all reagents being kept on ice. The volume of 1 M NaOH solution required to neutralize the acidic collagen stock solution was calculated by estimating the 70 mg/mL collagen stock to be 0.16 M acetic acid (AcOH). A 1:1.15 molar ratio of AcOH to NaOH was then used to estimate the volume of 1 M NaOH solution required to neutralize the AcOH. After mixing, the cold neutralized pregel solution was briefly centrifuged to eliminate large air bubbles. For scaffolds of 25, 50, and 75% (v/v) porosity, gelatin microparticles ∼30 μm in diameter (LifeSupport, Advanced Biomatrix) were rehydrated with cold deionized water, degassed in a vacuum chamber at room temperature for 30 min, and centrifuged at 2000 g for 5 min with the supernatant being discarded. The requisite volume of gelatin porogen for each condition’s respective porosity was pipetted into microcentrifuge tubes using a positive displacement pipet followed by adding 10× PBS in a 1:10 volume ratio of 10× PBS to total pregel solution volume into each microcentrifuge tube. Next, 70 mg/mL acidified collagen type I bioink solution was added to achieve a final pregel solution concentration of 10 mg/mL collagen with the remainder of the volume being deionized water. For example, 1 mL of a 50% porous collagen pregel solution consisted of 500 μL of porogen, 100 μL of 10× PBS, 142 μL of 70 mg/mL acidified collagen solution, and 258 μL of water. The volume of 1 M NaOH required to neutralize the AcOH was calculated similarly to the 0% porogen scaffolds.

Immediately after neutralization, 400 μL of pregel solution was then cast into a custom two-part mold using a positive displacement micropipette and incubated at 37 °C for 1 h to gel. The two-part mold was designed using computer-aided design (CAD) software (e.g., Autodesk Inventor; Autodesk Fusion 360), 3D-printed from poly(ethylene terephthalate) (PETG) with each half being bolted together using a single stainless steel M3 nut and bolt. The mold contained a central well 4 mm deep with walls that flared from 9 mm in diameter at the top to 12.5 mm at the bottom. The mold’s outer diameter of 34 mm allowed it to fit tightly into the wells of a 6-well plate.

After gelation of the collagen, 1× PBS was pipetted into the well and the scaffolds were incubated on a rocking plate (Thomas Scientific, 1154J70) at 30 rocks per minute at 37 °C overnight to remove residual gelatin from the sample to ensure all pores were open. For analysis, the custom two-part mold allowed for water immersion imaging within the 6-well plate. A z-stack image of each scaffold sample was captured using second harmonic generation (SHG) imaging on a multiphoton confocal microscope (Nikon A1R MP+) using a 16× (NA = 0.80) long working distance water immersion objective (Nikon). Every slice (*N* = 30) from each z-stack was analyzed in ImageJ (U.S. National Institutes of Health, https://imagej.net/ij/) by thresholding the image to generate a binary image of the collagen’s second harmonic signal versus the void produced by the vacated porogen. The percent surface area of the collagen signal was measured for each sample. The data was recorded and analyzed to produce a Pearson correlation coefficient to determine the correlation between the measured porosities.

### Dextran Transwell Diffusion Assay of Porous Collagen Scaffolds

Collagen scaffolds of increasing porosity were fabricated as described above, but with a modification at the casting stage where a positive displacement micropipette deposited pregel solution into 12-well Transwell inserts (Corning, 07-200-156). Scaffolds (*N* = 6 per porosity condition) were incubated at 37 °C for 1 h to gel. After gelation, 25 μL of 0.5 mg/mL 10 kDa FITC dextran (Thermo Fisher Scientific, D1821) was pipetted on top of each scaffold followed by periodic sampling of the 1× PBS well for 24 h, with an equal volume of 1× PBS being added to the well to maintain the same fluid level. Dextran concentrations were quantified using a spectrophotometer and a standard curve.

### Cell Culture

C2C12 myoblasts (ATCC, CRL-1772) were cultured based on previously described methods.^11,19,20^ Briefly, the cells were expanded at 37 °C under 5% CO2 in Dulbecco’s modified Eagle’s medium (DMEM) (Corning, 15-013-CM) supplemented with 10% (v/v) fetal bovine serum (FBS) (VWR, 89510-186), 1% (v/v) l-glutamine (Life Technologies, 25030081), and 1% (v/v) penicillin–streptomycin. Media was changed every 2 days and cells were passaged before reaching 80% confluency. For passaging, the cells were incubated with 0.25% Trypsin-EDTA for 5 min, resuspended in a 2:1 ratio of cell media to trypsin, and centrifuged at 180*g* for 5 min. The supernatant was aspirated, and the cells were resuspended in fresh medium before being transferred to new culture flasks. For cellularized scaffolds, the cells were lifted as described for passaging and then added to the pregel collagen and porogen solution at the stated cell concentrations.

### Fabrication and Analysis of Cellularized Porous Collagen Scaffolds

To assess the diffusion distance of nutrients within the porous scaffolds and the effect on cell viability, cellularized scaffolds were cast within the two-part PETG molds to control the interface with the surrounding media. The molds were sonicated for 30 min in sterile-filtered 70% ethanol, dried for 1 h in a biosafety cabinet, and then sterilized by 15 min UV ozone treatment. The mold halves were then bolted together using a single stainless steel M3 nut and bolt and placed into a 6-well plate. We compared 0 and 75% porous constructs with final concentrations of 5 mg/mL collagen, 1× PBS, 5 mM HEPES, and 40 × 10^6^ cells/mL. Using a positive displacement micropipette, 400 μL of pregel solution (*N* = 3 per condition) was then cast into the central well of the mold and incubated at 37 °C for 1 h to gel. Media was added to the well to cover the scaffolds in the molds and the plates were rocked at 30 rocks per minute at 37 °C with media changed every 2 days and samples analyzed at 0, 5, and 10 days. At each time point, molds were removed from the 6-well plate, the mold halves were slightly loosened, and a razor blade was inserted to section the tissue in half. The newly exposed faces were then stained with Live/Dead stain (L3224, Life Technologies) and incubated at 37 °C for 30 min. Scaffolds, still in their mold halves, were inserted into a custom 3D-printed holder within a bath of 1× PBS to orient the mold and cut scaffold face up and keep the cells submerged during imaging. A 12 mm glass coverslip was placed on the surface to flatten the exposed scaffold surface and facilitate imaging. Tile scans and 3D z-stack images of scaffolds were acquired using a 16× (NA = 0.80) long working distance water immersion objective (Nikon) on a Nikon A1R MP+ multiphoton confocal microscope. Images were analyzed in ImageJ using a custom macro which counted the number of live and dead cells within certain binned depths from the construct surface to produce viability as a function of depth for each day and porosity.

### Fabrication and Analysis of Perfused Porous Collagen Scaffolds

To assess the interaction between scaffold porosity and fluidic perfusion, we cast the cellularized porous collagen scaffold around a 3D bioprinted collagen tube within a perfusion bioreactor, adapted from previously published methods.^11^ First, we FRESH printed an acellular collagen-based scaffold consisting of a central vessel with an inner diameter (ID) of 1.4 mm and an outer diameter (OD) of 2.3 mm with a wall thickness of 450 μm spanning a 10 × 10 × 10 mm^3^ well.^11,21^ Scaffolds were printed using a 150 μm ID needle (Jensen Global, JG30–0.5HPX) with a 60 μm layer height, 3 perimeters, 35% infill density, and a speed of 23 mm/s. A 23 mg/mL acidified collagen type I bioink was prepared as previously described,^11,18,22^ by diluting 35 mg/mL acidified collagen bioink (LifeInk 240, Advanced Biomatrix, 5267) with deionized water. The collagen bioink was then loaded into a 2.5 mL gastight syringe with care to avoid the inclusion of bubbles in the ink. The syringe was loaded into a Replistruder syringe adapter which was then mounted onto a custom-designed 3D bioprinter.^23,24^ Sterile FRESH support bath (LifeSupport, Advanced Biomatrix) was reconstituted using a 4 °C sterile solution consisting of 100 mM HEPES, pH 7.4, and 1% (v/v) penicillin–streptomycin (Life Technologies, 15140-122). The support was then vortexed, degassed in a vacuum chamber for 1 h, and centrifuged at 3000*g* for 5 min. The supernatant was discarded, and the sterile support bath was transferred into a container for FRESH printing. Upon completion of printing, constructs were incubated at 37 °C for at least 30 min or until the support bath was entirely melted. The molten gelatin support bath was serially exchanged with warm print storage solution (PSS) which consisted of 1× phosphate-buffered saline (PBS), 50 mM HEPES, pH 7.4, and 1% (v/v) penicillin–streptomycin. Prints were incubated overnight in PSS to facilitate the removal of residual molten gelatin from the printed vessel networks. The next day, all constructs were subjected to 15 min UV ozone treatment (Novascan, PSD Pro) and incubated at 37 °C until use.

For the formation of the tissue construct and subsequent perfusion, the bioreactor was designed using CAD software and 3D-printed using Biomed Clear resin (Formlabs, RS-F2-BMCL-01) on a stereolithography (SLA) printer (Form 3B, Formlabs, RS-F2-BMCL-01) with 100 μm layers. Internal channels were flushed with 100% isopropyl alcohol (IPA) to purge resin from the channels. All parts were then sonicated (Branson, 3510) for 30 min in 100% IPA and dried. All parts were then baked in a UV oven (Creative CADWORKS, CureZone MKII) for 5 min. All 3D-printed parts were sonicated for 30 min in sterile-filtered enzymatic detergent (Enzol, Johnson & Johnson), washed 3 times with sterile DI H_2_O, sonicated for 30 min in sterile-filtered 70% ethanol, dried for 1 h in a biosafety cabinet, and then sterilized by 15 min UV ozone treatment. Next, the collagen vessel scaffold was placed in the bioreactor and C2C12s in collagen gel with porogen were cast around the printed vessel. The casting was similar to that previously described with the gelatin porogen being rehydrated in a media-based solution instead of deionized water. The final composition cast around the collagen vessel was 75% (v/v) porogen, 20 × 10^6^ cells/mL, and 12 mg/mL collagen type I. A control construct without porogen was cast with similar cell and collagen concentrations. After casting, the bioreactor was sealed, placed in an incubator, and perfused at 220 μL/min using a peristaltic pump (Ismatec, EW-95663-34) for 10 days or cultured statically as control, with media changed every 2 days. For analysis, tissue constructs were sectioned in half using microdissection scissors (Dr Instruments, 9M) and stained for viability using LIVE/DEAD. Tile scans and 3D z-stack images were acquired using a 4× (NA = 0.20) Plan Apochromat objective (Nikon) on a Nikon A1R MP+ multiphoton confocal microscope. Images were analyzed in ImageJ using a custom macro to count the number of live and dead cells as a function of radius from the vessel center. The distance from the vessel center where cell viability dropped below 75% was determined to be the radius of viability (ROV) of the vessel.

### Computational Model of Oxygen Diffusion within Porous Collagen Scaffolds

To better understand the changes in oxygen concentration and cellular consumption as a function of scaffold porosity and distance from the surface, a steady-state numerical model was developed in COMSOL Multiphysics 6.2 using the finite element method (FEM). A 2D geometry of the cross-sectional area of the cell-laden porous scaffold was implemented with oxygen diffusion being governed by the generic diffusion equation (eq 1):
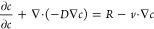
1where *c* is the concentration of oxygen [mol/m^3^], *D* is the diffusion coefficient of oxygen within the porous collagen [m^2^/s], *R* is the reaction rate of oxygen [mol/(m^3^·s)], and *v* is the velocity field (set to 0 m/s).^25^ C2C12 oxygen consumption was assumed as Michaelis–Menten-type kinetics^26^ with a *R*_max_ = 0.22 fmol/(min·cell),^27^ a Michaelis–Menten constant *K*_MM_ = 0.00133 mM,^28^ and a cell density (CD) of 20 × 10^6^ cells/mL (eq 2).
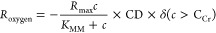
2

To account for necrosis and prevent negative oxygen concentrations, a step-down function (δ) was added to shut off oxygen consumption below *C*_Cr_ = 1.45 × 10^–4^ mM, a value where skeletal muscle is shown to have compromised metabolic activity equaling 0.1 mmHg of oxygen.^29,30^ A boundary condition of atmospheric oxygen concentration, *c* = 0.2 mM,^29^ relating to 140 mmHg, was added to one edge of the domain to act as a constant oxygen source simulating perfusion. An initial oxygen concentration at this value was also applied throughout the domain. To account for the porogen within the collagen scaffold, the diffusion coefficient of oxygen was calculated from the volume fraction of the porogen multiplied by the diffusion coefficient of oxygen in media (assumed equal to oxygen in water), *D*_O_2_,w_ = 3.0 × 10^–9^ m^2^/s,^29^ added to the volume fraction of the 12 mg/mL collagen multiplied by the diffusion coefficient of oxygen in high-density collagen (11%), *D*_O_2_,col_ = 4.5 × 10^–10^ m^2^/s.^31^ Cell volume was assumed to be negligible. Due to the large size of the domain (10 mm × 3.85 mm) compared to the size of the gelatin porogen (30 μm), this approach models the porous scaffold as a homogeneous material by averaging the diffusion coefficients of oxygen in water and high-density collagen based on their respective volumetric proportions. However, it fails to account for any percolation created by the porogens that would ultimately help oxygen diffuse further into the scaffold.

### Statistical Analysis

Statistical analysis was performed using Prism 9.3 software (GraphPad Software), and unless stated otherwise, each experiment was performed in triplicate. Transwell dextran diffusion was analyzed using a two-way ANOVA followed by Tukey multiple-comparisons post test. The effect of tissue porosity on C2C12 viability was analyzed using *t* tests. The effect of perfusion on C2C12 tissue viability was analyzed using *t* tests. Statistical significance was based on a *P* < 0.05 (*) with lower *P*-values being denoted as *P* < 0.01 (** or &), *P* < 0.001 (*** or %), and *P* < 0.0001 (**** or #). Nonsignificant *P*-values were denoted as ns.

## Results and Discussion

### Controlling Collagen Scaffold Porosity via Gelatin Microparticle Porogen

**Figure 1:**
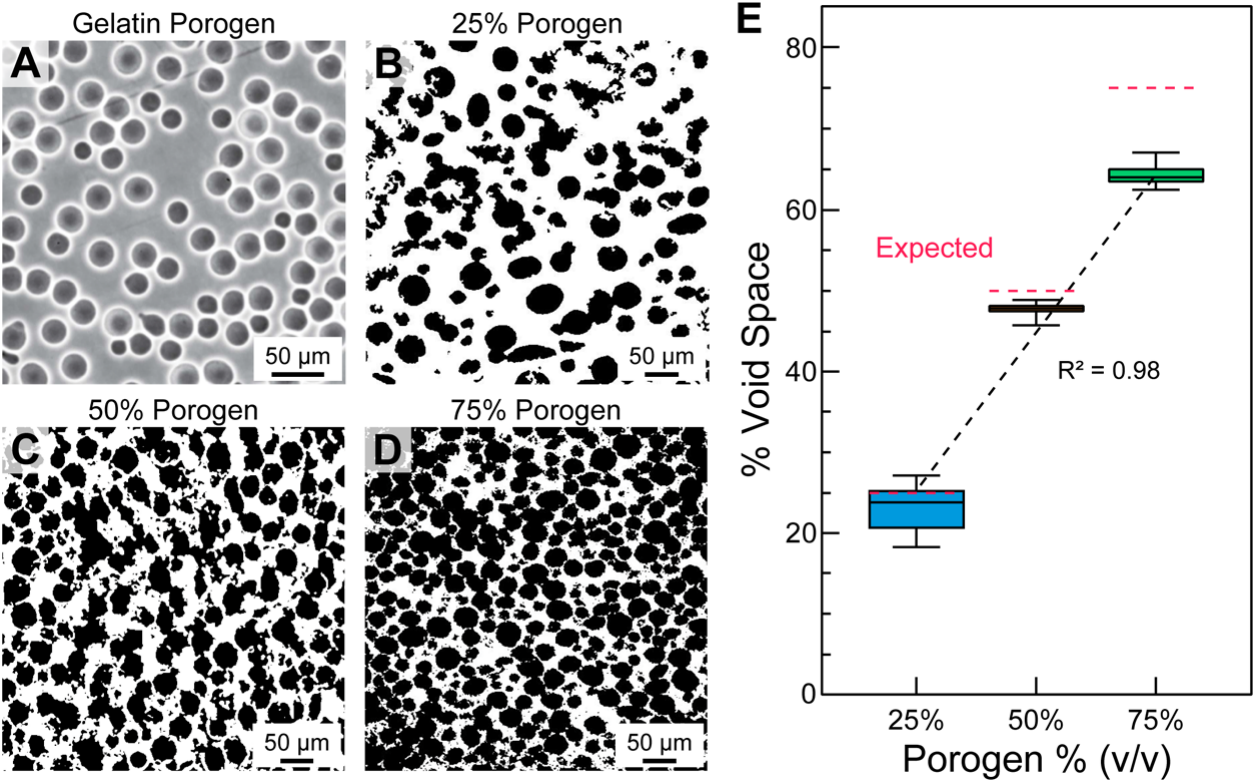
Controlling collagen scaffold porosity via gelatin microparticle porogen. (A) Representative phase microscopy image of the gelatin microparticle porogen in PBS. (B–D) Collagen scaffolds with various porosities imaged by second harmonic imaging using multiphoton microscopy and then converted to a binary image with the pores in black and collagen in white. (E) Analysis of percent void space for each porous tissue showing a high degree of correlation between expected and measured percent void space (*N* = 30).

To create the porous collagen scaffolds, gelatin microparticles were mixed in with the neutralized collagen solution, allowed to gel at 37 °C, and then the gelatin microparticles were melted out to create the micropores. The gelatin microparticles used were repurposed LifeSupport, which is a commercially available version of the support bath material used for FRESH 3D bioprinting.^11^ This choice was made because the LifeSupport comes as a sterile, lyophilized powder that after rehydration in sterile buffer results in gelatin microparticles with a diameter of ∼30 μm (Figure 1A). Incorporating the gelatin microparticles in volume ratios of 0, 25, 50, and 75% (v/v) produced collagen scaffolds with comparable levels of void space, as imaged by second harmonic imaging using multiphoton microscopy (Figure 1B–1D). Quantitative image analysis of the scaffold images showed a linear relationship between the volume of porogen added and the measured area fraction of void space (Figure 1E). Another reason LifeSupport was chosen as the porogen is because the microparticle diameter of ∼30 μm is close to the 30–40 μm diameter found to promote microvascular growth, and healing through mechanisms such as M2 macrophage polarization.^6,8,32^ These results confirm the ability to tailor scaffold porosity over a wide range, with the highest volume ratio of 75% appearing to result in a high level of pore interconnectivity not observed at lower porogen volume ratios.

### Collagen Scaffold Porosity Increases Molecular Diffusivity

**Figure 2:**
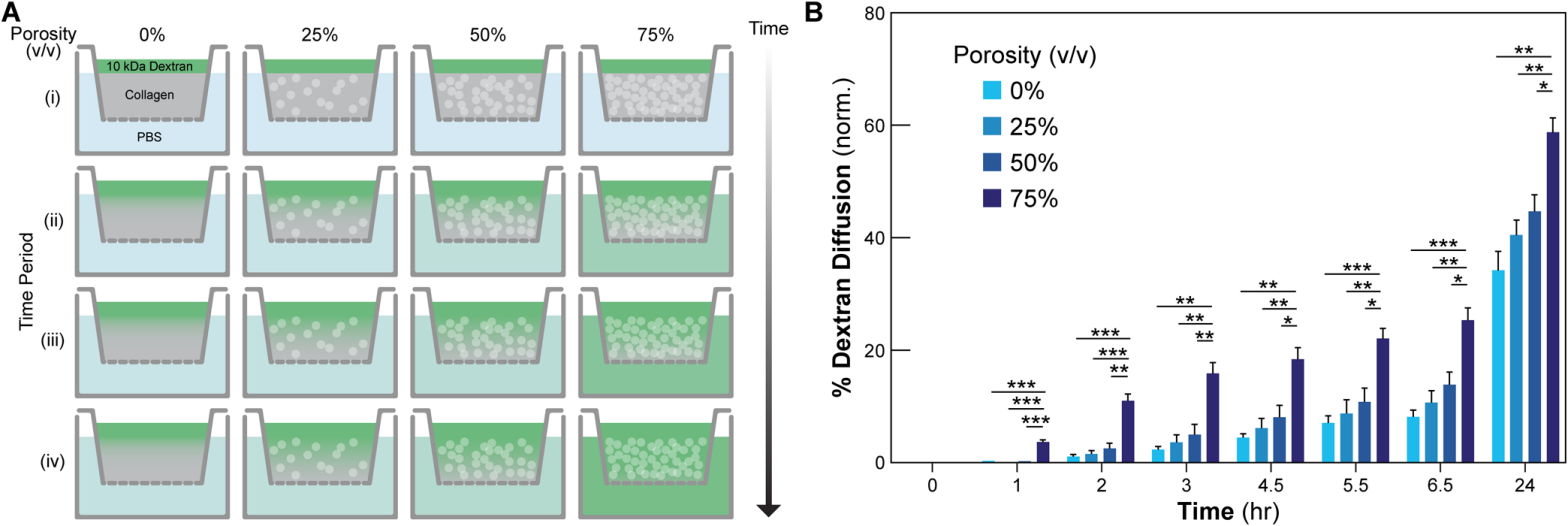
Dextran transwell diffusion assay of porous scaffolds. (A) Schematic of the experimental setup showing collagen scaffold (gray) with 0–75% (v/v) porosity gelled inside Transwell inserts. The collagen acts as a barrier between the 10 kDa FITC dextran (green) on the surface and the PBS (blue) in the Transwell below. Fluid samples from the well are taken over the course of 24 h (i–iv) for spectrophotometric analysis. (B) Comparison of dextran diffusion for each scaffold type and analysis time point. The percentage of dextran that diffused was normalized to the total concentration in the system, where a value of 100% would indicate that the dextran concentration in and below the Transwell had equalized. [*N* = 6, data are means ± SEM, *P < 0.05 (two-way ANOVA followed by Tukey multiple-comparisons post test)].

Porosity has an impact on diffusion rates because oxygen and molecules will diffuse faster through the media-filled pores than the collagen type I that composes the rest of the scaffold. To quantify the effect of matrix porosity on diffusivity, fluorescent 10 kDa dextran was placed on top of collagen gels with porosity from 0 to 75% (v/v) in a Transwell plate experiment and allowed to diffuse through the scaffolds into the media reservoir below (Figure 2A). The 10 kDa dextran was selected due to its molecular size and diffusion properties, which are representative of glucose and small growth factors and are critical for C2C12 myoblast viability and functionality. Spectrophotometric analysis confirmed a statistically significant increase in diffusion with increasing porosity across all time points (Figure 2B). Notably, the 0, 25, and 50% porosity samples appeared to increase in diffusion linearly with porosity. However, there was a larger-than-expected increase in diffusion for the 75% porosity samples. This is thought to be due to the occurrence of a critical porosity in the collagen scaffold, where pore density is sufficient to be interconnected and allow for the continuous percolation of fluid through the scaffold, significantly increasing the rate of diffusion. Based on these results, the 75% porosity scaffold was identified as the optimal condition to maximize diffusion and thus cell viability once cells are introduced into the constructs.

### Increased Molecular Diffusivity of Porous Scaffolds Improves Cell Viability

**Figure 3:**
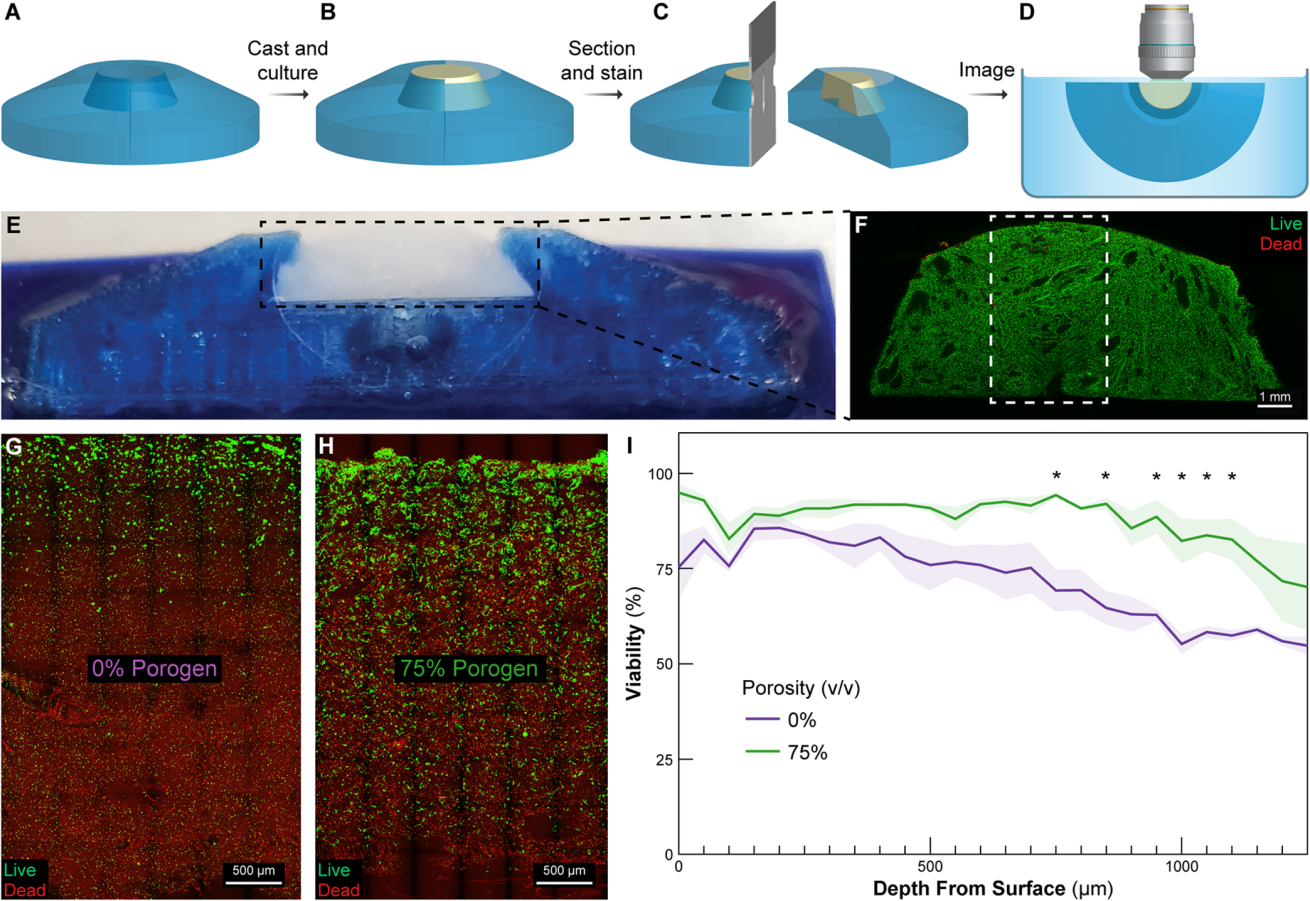
Analyzing the effect of scaffold porosity on cell viability. (A) Schematic of the two-part 3D-printed mold. (B) Cellularized constructs of 0–75% (v/v) porogen are cast into the molds, placed into 6-well plates, and cultured on a rocking plate for up to 10 days. (C) The two-part mold is loosened, and a razor blade is inserted to section the tissue without deformation. (D) The sectioned tissues are incubated in LIVE/DEAD stain and imaged. (E) A construct at day 0 after fabrication in the two-part mold after sectioning in one-half. (F) A confocal LIVE/DEAD image of (E) at day 0. (G, H) Day 5 LIVE/DEAD confocal images of (G) 0% porogen and (H) 75% porogen constructs from the same central region in (F, white box) showing increased viability for 75% porous constructs. (I) Day 10 cell viability as a function of depth from the apical surface of either 0 or 75% porous constructs [*N* = 3, data are means ± SEM, *P < 0.05 (multiple *t* test)].

Next, cells were integrated into the collagen scaffolds to determine if the increased rate of diffusion for the 75% porosity condition translated into higher cell viability within thicker constructs. To test this, C2C12 myoblasts at 40 × 10^6^ cells/mL in 12 mg/mL collagen at 0% or 75% porosity were cast into custom plastic molds 3D-printed from PETG (Figure 3A,B). These molds restrict media access to the apical surface of the scaffolds, with diffusion proceeding from this surface down into the bulk construct. The two-part molds were designed to fit into a 6-well plate and the two halves could be separated slightly to allow for in situ vertical sectioning of casted constructs (Figure 3C). By imaging the exposed vertical section and staining with LIVE/DEAD, cell viability as a function of depth from the apical surface exposed to media during culture could be assessed via confocal microscopy (Figure 3D). Analysis of a construct immediately after fabrication showed that the scaffold could be successfully sectioned with this approach (Figure 3E) and that cells were viable and alive throughout the entire volume (Figure 3F). At day 5 of culture, cell viability for 0% porous constructs (Figure 3G) dropped to <85% at a depth of ∼200 μm from the tissue surface, a value frequently seen throughout the literature.^5,7,33−38^ Viability for 75% porous constructs remained >85% until a depth of ∼1 mm from the apical surface (Figure 3H), demonstrating the increase in viability that the microporosity enables. Culturing for longer at 10 days to ensure that cells deprived of nutrients were no longer alive produced similar results, with significantly higher viability for porous tissues ≥ 750 μm from the surface (Figure 3I). These results suggest that the observed increase in diffusivity of the microporous constructs results in an increase in nutrient diffusion that significantly improves cell viability over extended culture periods.

### Perfusing Porous Scaffolds with Vascular-like Channels Improves Cell Viability and Minimizes Necrotic Core Formation

**Figure 4:**
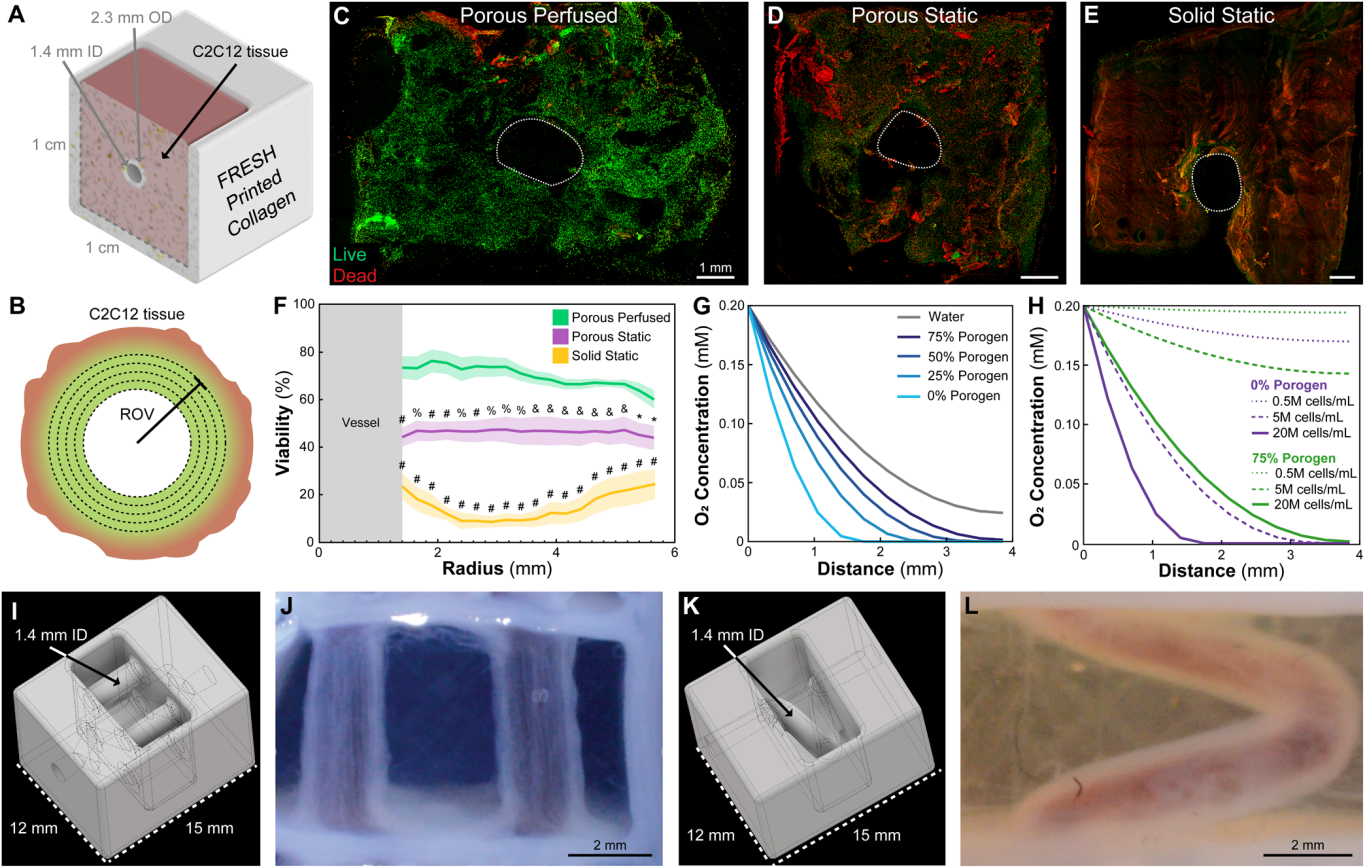
Perfusion of single-vessel scaffolds improves cell viability and minimizes necrotic core formation. (A) Schematic of the single-vessel scaffold containing a C2C12-laden collagen gel (pink) sectioned in half. (B) Illustration of the radius of viability (ROV) calculation where cell viability as a function of radius. The ROV is deemed as the radius at which viability drops <75%. (C–E) Confocal images of the sectioned C2C12 tissue faces stained with LIVE/DEAD after being cultured under perfused (C) or static (D, E) conditions for 5 days. (F) Percent viability as a function of radius from the vessel center [*N* = 6, data are means ± SEM, *P* < 0.05 (*) with lower P-values being denoted as *P* < 0.01 (&), *P* < 0.001 (%), and *P* < 0.0001 (#) (shown comparisons are in relation to the porous perfused condition) (two-way ANOVA followed by Tukey multiple-comparisons post test)]. (G) Plot of oxygen concentration as a function of depth from the collagen tube into the scaffold based on an FEM simulation comparing different porosities at 20 M cells/mL. (H) Plot of oxygen concentration as a function of depth from the collagen tube into the scaffold based on an FEM simulation comparing different cell densities for the 0 and 75% porogen conditions. (I) Schematic of a 4-vessel scaffold with a single-inlet and single-outlet design. (J) The 4-vessel scaffold FRESH 3D bioprinted with collagen and perfused with red dye to demonstrate patency. (K) Schematic of a helical vessel scaffold. (L) The helical vessel scaffold FRESH 3D bioprinted with collagen and perfused with red dye to demonstrate patency.

Having demonstrated that 75% porous scaffolds increase cell viability, we next investigated whether adding perfusion of larger (1 cm^3^) constructs, where diffusion and convective flow are combined, would improve viability further compared to static cultured controls. To test this, we adapted our previously published single vascular-like channel scaffold design,^11,21,39^ where a FRESH 3D bioprinted collagen tube with 1.4 mm ID, 2.3 mm OD, 450 μm wall thickness, and 1 cm length was suspended through the middle of a 1 cm high and 1 cm wide volume (Figure 4A). Around the tube were cast the 0% (solid) or 75% porous scaffolds consisting of C2C12 myoblasts at 20 × 10^6^ cells/mL and 12 mg/mL collagen. After 10 days of culture, tissues were sectioned in half perpendicular to the vessel axis (in the radial direction) and stained with LIVE/DEAD. Representative confocal images of the 75% porous perfused (Figure 4C), 75% porous static (Figure 4D), and 0% porous (solid) static (Figure 4E) constructs showed the major qualitative difference in overall shape and cell viability between the conditions. Notably, the 75% porous and perfused construct showed viability throughout the construct and the scaffold itself had compacted over time in culture due to active cell remodeling of the collagen. This compaction behavior is a typical response for cells in collagen gels.^11,21,40^ In addition to the LIVE staining, this provides further evidence that the C2C12s are viable and functional because the compaction process requires actomyosin motor activity of stress fibers in the cytoskeleton.^41^ In contrast, the 75% porous and static construct showed notable regions of DEAD labeled cells and showed reduced compaction, demonstrating the benefit of convective flow from perfusion over diffusion alone. Finally, the 0% porous (solid) construct showed very low cell viability throughout and minimal compaction, essentially maintaining the same dimensions as at the start of the experiment. For quantitative analysis, a region of viability (ROV) was calculated using a custom ImageJ macro that counted the number of live and dead cells as a function of radius from the vessel center. The ROV was deemed to be the distance from the vessel center at which cell viability dropped below 75%. The ROV of the vessel was determined to be ∼4 mm from the center with statistically significant improvements to cell viability extending out to ∼5 mm (Figure 4F). Static cultured 75% porous constructs were consistently 50% viable throughout, while 0% porous (solid) static cultured constructs were ∼25% viable near the vessel and periphery and even less viable in between. For comparison, the ROV of similar tissue constructs perfused but without porosity, from previously published studies, was ∼1 mm, clearly demonstrating the synergistic impact that porosity and perfusion can have together.^11^

Implementing an FEM oxygen diffusion model provides additional understanding of how scaffold porosity influences oxygen distribution within the structure. The model assumes that the collagen tube is being constantly perfused with oxygenated media, resulting in a constant oxygen supply at the inner surface of the construct. The simulation results revealed a clear correlation between an increasing percentage of porogen and higher oxygen concentration deeper into the scaffold (Figure 4G). The model assumes that the porous collagen scaffold behaves as a homogeneous material, meaning that oxygen diffusion is primarily dictated by the effective diffusion coefficient. This effective diffusion coefficient determines how rapidly oxygen can diffuse through the scaffold before being consumed by the integrated cells. This relationship remained consistent over a range cell densities from 0.5 to 20 million cells per milliliter (Figure 4H). In reality, however, the porous nature of the scaffold introduces additional complexity. Oxygen diffusion is expected to be significantly higher in the cell culture media-filled pores compared to the dense collagen matrix itself, leading to heterogeneous transport dynamics. As a result, a more pronounced shift in oxygen concentration would be expected once the scaffold reaches its percolation threshold, where interconnecting pores allow for continuous diffusion pathways. While the model simplifies these complexities by treating the scaffold as a uniform material, it still provides valuable insights into oxygen availability and, consequently, C2C12 cell viability throughout the scaffold. This is particularly true at early time points when diffusion is the dominant mechanism for oxygen transport before cellular activity remodels the microenvironment.

Although the ROV of the 75% porous and perfused construct is significantly improved compared to controls, there are three major factors that contribute to this being a conservative ROV estimate. First, the base metabolic rate of C2C12 myoblasts is higher than other cell types such as endothelial cells,^42,43^ meaning the C2C12s will consume nutrients more rapidly than tissue constructs consisting of cells with slower metabolisms. Second, the ECM protein density of 12 mg/mL collagen is at least 4 times higher than that commonly used in tissue engineering studies, such as 1–10 mg/mL fibrin^35,44−50^ or 1–6 mg/mL collagen.^35,51−59^ This increased scaffold density will decrease the rate of diffusion compared to scaffolds with lower ECM protein density. Third, the C2C12 density of 20 × 10^6^ cells/mL is considerably higher than that commonly used in tissue engineering, which is in the range of 0.5–10 × 10^6^ cells/mL.^35,44,45,47,52,53,57,58,60,61^ This suggests that the size and spacing of engineered vessels need to be designed with respect to the cell type, cell density, and matrix composition. For example, had the ROV of a single vessel been 2 mm, a total of four equally spaced vessels would be required to maintain the viability of a similar-sized construct (Figure 4I,J). Conversely, by increasing the diffusivity of a tissue, single-vessel scaffold with a helical shape could have a larger ROV surrounding it (Figure 4K,L), providing nutrients to a significant volume of a construct before a microvascular network has formed throughout the engineered tissue. The oxygen transport model demonstrates that combining 75% porosity with perfusion extends the radius of viability (ROV) up to 3 mm for a cell density of 20 M cells/mL and to >4 mm for lower cell densities (Figure 4H). These findings underscore the critical interplay between scaffold porosity and convective flow in enhancing tissue viability. Additionally, the model provides a framework for optimizing vessel design and spacing to maximize nutrient delivery and minimize necrotic regions while scaling up tissue constructs to clinically relevant dimensions.

## Conclusions

In conclusion, this study demonstrates the efficacy of combining microporosity and perfusion strategies to address the critical challenge of nutrient transport in engineered tissue constructs. By incorporating gelatin microparticles as a thermoresponsive porogen, we achieved a 75% porous collagen scaffold, which significantly enhanced nutrient diffusivity and improved cell viability compared to nonporous constructs. When integrated with FRESH 3D bioprinting to create perfused vascular-like channels, these porous scaffolds supported sustained cell viability deep within large tissue volumes, reducing the formation of necrotic cores. Notably, even under conditions of high cell density and collagen concentration, the synergistic effects of porosity and perfusion extended the radius of viability, suggesting that these methods can be tailored to support the growth of more metabolically demanding tissues. This dual approach offers a promising path forward for engineering thicker and more complex tissues by alleviating the constraints of passive diffusion and slow vascular ingrowth. Future work will explore scaling these strategies to larger constructs and incorporating additional cell types, ultimately bringing engineered tissues closer to clinical application.
